# Supplementary material for: Strengthening health system’s capacity for linkage to HIV care for adolescent girls and young women and adolescent boys and young men in South Africa (SheS’Cap-Linkage): Protocol for a mixed methods study in KwaZulu-Natal, South Africa
Source: PLoS One. 2023 Feb 13;18(2):e0271942. doi: 10.1371/journal.pone.0271942 (PMC9925067; doi:10.1371/journal.pone.0271942)
Supplement: S1 Table — (DOCX) [file pone.0271942.s001.docx]

**Supplement 1: Socio-Demographic characteristics of the HIV-positive participants at baseline and linkage in care in the first month of follow-up**

| ***Variable*** | ***Total*** | | | ***Linked in care (LiC)*** | | | ***Not linked in care (nLiC)*** | | |  | |
| --- | --- | --- | --- | --- | --- | --- | --- | --- | --- | --- | --- |
|  | ***n*** | ***%*** | ***95% CI*** | ***n*** | ***%*** | ***95% CI*** | ***n*** | ***%*** | ***95% CI*** | **F-value** | **p-value** |
| Nationality |  |  |  |  |  |  |  |  |  |  |  |
| Facility type |  |  |  |  |  |  |  |  |  |  |  |
| Sex |  |  |  |  |  |  |  |  |  |  |  |
| Ethnicity |  |  |  |  |  |  |  |  |  |  |  |
| Education level |  |  |  |  |  |  |  |  |  |  |  |
| Age, median (IQR) |  |  |  |  |  |  |  |  |  |  |  |
| Marital status |  |  |  |  |  |  |  |  |  |  |  |
| Access to US$14 in emergencies |  |  |  |  |  |  |  |  |  |  |  |
| Received child support grant |  |  |  |  |  |  |  |  |  |  |  |

p-value of$\leq0.05$ considered statistically significant

p-values derived using Mann Whitney U-test for continuous data

p-values derived using Chi-squared test considering the cluster design

proportions (%) for the columns reported as n/N and the associated 95% CI
