# Supplementary material for: Strengthening health system’s capacity for linkage to HIV care for adolescent girls and young women and adolescent boys and young men in South Africa (SheS’Cap-Linkage): Protocol for a mixed methods study in KwaZulu-Natal, South Africa
Source: PLoS One. 2023 Feb 13;18(2):e0271942. doi: 10.1371/journal.pone.0271942 (PMC9925067; doi:10.1371/journal.pone.0271942)
Supplement: S2 Table — (DOCX) [file pone.0271942.s002.docx]

**Supplement 2: Factors influencing linkage to care after the first month of HIV-positive diagnosis**

| **Variable** | **N (%)** | **Unadjusted model**  **OR (95% CI)** | **p-value** | **Adjusted model**  **aOR (95% CI)** | **p-value** |
| --- | --- | --- | --- | --- | --- |
| ***Socio-demographic variables*** | | | | | |
| Type of facility |  |  |  |  |  |
| Age in years |  |  |  |  |  |
| Highest education attained |  |  |  |  |  |
| Sex |  |  |  |  |  |
| Marital status |  |  |  |  |  |
| Work in the past 12 months |  |  |  |  |  |
| Received child support |  |  |  |  |  |
| Received a disability grant |  |  |  |  |  |
| ***Enablers and Barriers*** | | | | | |
| Transport used to get to facilities |  |  |  |  |  |
| Time to get to the facility |  |  |  |  |  |
| Condom use in the last sexual experience |  |  |  |  |  |
| Condom use with once-off partner |  |  |  |  |  |
| Reason for HIV testing |  |  |  |  |  |
| Physical Partner Violence from male partner |  |  |  |  |  |
| Area of residence |  |  |  |  |  |

CI – Confidence Interval; OR – Odd ratio; aOR – adjusted Odds Ratio (all derived using logistic regression)

p-value of$\leq0.05$ considered statistically significant
